# The Effect of Thymoquinone and Platelet-Rich Plasma on Intra-Abdominal Adhesions

**DOI:** 10.3390/medicina61071119

**Published:** 2025-06-20

**Authors:** Gökhan Karaca, Hakan Amioğlu, Mevlüt Recep Pekcici, Huri Demirci

**Affiliations:** 1 Department of General Surgery, Faculty of Medicine, Sincan Training and Research Hospital, Health Science University, Ankara 06949, Turkey; hakanamioglu@gmail.com; 2 Department of General Surgery, Ankara Education and Research Hospital, Ankara 06230, Turkey; pekcici@yahoo.com; 3 Department of Medical Biochemistry, Faculty of Medicine, Biruni University, Istanbul 34295, Turkey; huridedeakay@gmail.com

**Keywords:** adhesion, inflammation, platelet-rich plasma, thymoquinone

## Abstract

*Background and Objectives:* At present, intra-abdominal adhesions (IAAs) continue to be an important problem in surgery due to morbidity and mortality risks. Thymoquinone (TQ) and platelet-rich plasma (PRP) are molecules with known anti-inflammatory and antioxidant effects. However, a limited number of studies have investigated their efficacy in IAAs. In this study, we aimed to demonstrate the efficacy of TQ and PRP in reducing the development of IAAs and determine which molecule is more advantageous using an experimental animal model. *Materials and Methods:* Fifty-five male Wistar albino rats were included in the study. Five rats were used to obtain PRP, while fifty rats were randomly assigned to five groups (n = 10 per group): group I (sham) did not receive any treatment; group II (control) received no treatment after a cecum hemorrhage procedure; group III (saline) received 1 mL of saline treatment around the cecum after hemorrhage; group IV (PRP) received 1 mL of PRP (containing 3 × 10^6^ platelets/mL) around the cecum after hemorrhage; and group V (TQ) received 1 mL of TQ (containing 2 mg/mL TQ) around the cecum after hemorrhage. On the 10th day, IL1-β, TNF-α, E-selectin, and P-selectin levels were measured from the blood serum samples, and the cecum was histopathologically evaluated. *Results:* The lowest adhesion formation in terms of biochemical parameters was obtained in the TQ group (*p* < 0.05). Histopathological evaluations showed that saline, PRP, and TQ treatments were all effective, but none was superior. *Conclusions:* When histopathologically evaluated, saline, TQ, and PRP have similar effects in IAAs. However, when evaluated in terms of biochemical parameters, TQ prevented the formation of intra-abdominal adhesions more effectively than saline or PRP, owing to its strong anti-inflammatory and antioxidant properties.

## 1. Introduction

Intra-abdominal adhesions (IAAs) are a critical problem during surgery because they are difficult to prevent and treat, resulting in morbidity and mortality risk. Surgical operations are the most common cause of intra-abdominal adhesions, but other causes can be attributed to inflammatory diseases and the presence of foreign bodies [1,2]. To date, many studies have described potential means of reducing postoperative intra-abdominal adhesions, such as changing the viscosities of the fluids put in the peritoneal cavity, using mesenchymal stem cells, and using Ankaferd BloodStopper products [3,4,5]. The aims of those studies varied from reducing inflammation to increasing fibrin lysis to preserving serosa, but their applications in clinical practice were limited.

Thymoquinone (TQ) is the active ingredient of the volatile oil of Nigella sativa (blackseed) and has been proven to have anti-inflammatory and antioxidant effects [6]. Houghton and colleagues showed that N. sativa fixed oil significantly inhibited the cyclooxygenase and 5-lipoxygenase pathways of arachidonate metabolism in rat peritoneal leukocytes by inhibiting the formation of thromboxane B2 (TXB2) and leukotriene B4 (LTB4) metabolites [7]. TQ also has a scavenging activity against superoxide anions, hydroxyl (OH−) radicals, and singlet molecular oxygen [7,8,9,10]. Modulation of NF-κB and TNF-α by TQ attenuates proinflammatory and oxidative responses [8].

Platelet-rich plasma (PRP) contains many growth factors, such as PDGF, TGF-ꞵ, IGF-1, and VEGF. When the platelets of PRP are activated, all of these factors are released into the environment and exert biological effects [11,12]. Many studies have shown that PRP increases wound healing and tissue regeneration, especially in orthopedic surgery, dentistry, and plastic surgery [13,14,15,16].

Selectins and cell surface glycoproteins are members of the adhesion molecule family and are involved in the capturing, rolling, and slow-rolling stages of PMLs [17,18]. Evidence from animal studies indicates that P-selectin plays a central role in the binding of neutrophils to the activated endothelium in the early stage as well as in the spontaneous rolling of neutrophils within the post-capillary venules [18]. E-selectin is found in endothelial cells and is rapidly released from these cells in response to inflammatory cytokines [17,18]. Selectin levels are associated with the progression of the inflammatory process [17]. Therefore, we selected these molecules as indicators of an inflammatory response.

TNF-α and IL1 are additional main causes of IAAs. In many studies, TNF-α, IL1-β, TGF-β, and IFN-γ have been shown to increase adhesion by increasing the number of cell adhesion molecules [19,20,21,22]. To evaluate adhesion, we investigated TNF-α and IL1-β levels.

Studies investigating the effects of PRP and TQ on intra-abdominal adhesions are rare. In this study, we aimed to investigate the potential effects of TQ and PRP on intra-abdominal adhesions, as well as whether TQ or PRP is superior during treatment, based on their previously proven anti-inflammatory activity. We biochemically and histopathologically evaluated TNF-α, IL1-β, E-selectin, and P-selectin levels for the assessment of adhesion development.

## 2. Materials and Methods

This study was conducted with approval from the Animal Ethics Committee of Kırıkkale University (2014/12). All the study protocols were performed in accordance with the Declaration of Helsinki, the Animal Welfare Act, and the Guide for the Care and Use of Laboratory Animals. Fifty-five male Wistar rats with a mean weight of 240–280 g were used in the study. To determine an appropriate sample size, we used articles published in the literature that included a similar experimental model as a reference, and, therefore, no power analysis was applied. All rats were fed with standard feed and water. Five rats were used to obtain PRP, while the other fifty rats were divided into five groups, each containing ten rats.

All rats were anesthetized intraperitoneally with ketamine 90 mg/kg (Ketas, 500 mg/10 mL Pfizer Inc., Berlin, Germany) and xylazine 10 mg/kg (Rompun, Bayer, Leverkusen, Germany). Rats were divided into 5 groups as follows:

Group I (n = 10): This is the sham (S) group. No surgical procedures (or other treatments) were conducted on group I.

In the other 4 groups, following anesthesia, field cleaning was performed with povidone-iodine, and the abdomen was opened with a midline incision. After the cecum was exposed, dry gauze was rubbed on it until petechial hemorrhages were observed [17].

Group II (n = 10): This is the control (C) group. Group II did not receive any treatment.

Group III (n = 10): This is the saline (SF) group. A total of 1 mL of saline was applied around the cecum.

Group IV (n = 10): A total of 1 mL of PRP (containing 3 × 10^6^ platelets/mL) was applied around the cecum.

Group V (n = 10): A total of 1 mL of TQ diluted with saline (containing 2 mg/mL TQ) was applied around the cecum. TQ was obtained from Sigma Aldrich (St. Louis, MO, USA).

All rats were euthanized with high-dose anesthesia on the 10th day of the operation. The abdomen was opened with an inverted U incision, allowing adhesions to be observed. After taking a blood sample for biochemical investigations, the ileum and cecum were excised and taken out of the abdomen.

### 2.1. PRP Preparation

Five male Wistar albino rats weighing 220–240 g were used for the production of PRP. Blood samples were taken from previously anesthetized rats by cardiac puncture and placed in tubes containing 3.2% sodium citrate (Merck, Darmstadt, Germany) with a blood/citrate ratio of 9/1. Blood samples were centrifuged for 10 min under 400 G. The supernatant remaining at the top of the tube was centrifuged for an additional 10 min under 800 G. Two thirds (the upper part) of the material in the tube was discarded and one third (the lower part) was kept at a temperature of −20 °C until needed [23].

### 2.2. Histopathological Examination

The cecum and ileum were excised completely and taken out of the abdomen. The tissue samples were stored in 10% formalin solutions. After processing the tissue, the samples were embedded in paraffin blocks. Then, 5 μm thick sections of the paraffin blocks were prepared and stained using hematoxylin–eosin and Mason’s Trichrome. All samples were evaluated according to Zühlke’s microscopic adhesion classification by the same pathologist, who did not know which group each sample was from (i.e., the pathologist was blind to the samples) [24] (Table 1).

### 2.3. Biochemical Analysis

Blood samples collected from rats were centrifuged at 3000× *g* for 10 min to obtain blood serum. Serum samples were stored at −80 °C until calculation for the levels of E-selectin, P-selectin, TNF-α, and IL1-β. The levels were determined by an ELISA plate reader (Thermo Scientific Multiskan FC, 2011-06, Waltham, MA, USA). All samples in the study were measured in duplicate.

### 2.4. ELISA Kit

Samples were thawed, and enzyme immunoassay kits (Rat E-Selectin ELISA kit, cat num CKE11636, Eastbiopharm Co., Ltd., Hanngzhou Assay, Haungzhou, Zheijiang, China; Rat P-Selectin ELISA kit, cat num CKE11638, Eastbiopharm Co., Ltd., Hanngzhou Assay; Rat TNF-alfa ELISA kit, cat num CKE11540, Eastbiopharm Co., Ltd., Hanngzhou Assay; Rat IL-1 beta ELISA kit, cat num CKE10078, Eastbiopharm Co., Ltd., Hanngzhou Assay) were used to quantitatively measure serum samples. Briefly, samples and standards were added to the monoclonal antibody enzyme well, which was pre-coated with related anti-rat monoclonal antibodies. After incubation, biotin was added to all wells and combined with streptavidin–HRP conjugates to form an immune complex. Then, this complex was incubated and washed to remove the uncombined enzyme. Chromogen Solutions A and B were added to make the color of the liquid change to blue. Due to the effects of adding an acid, the color finally became yellow. Optical density was read on a standard automated plate reader at 450 nm (Thermo Scientific Multiskan FC (2011-06), Waltham, MA, USA). The ELISA kits employed in this study exhibited detection ranges of 5–1000 ng/L for TNF-α, 20–800 ng/L for IL-1β, 1–350 ng/mL for E-Selectin, and 0.5–200 ng/mL for P-Selectin.

### 2.5. Statistical Analysis

For statistical analysis, microscopic adhesion classification grades were organized by numbers (i.e., grade 0 = 0; grade 1 = 1; grade 2 = 2; grade 3 = 3; grade 4 = 4). All of the results are reported as means ± standard deviations of the mean. Statistical analysis was performed using SPSS© v 16 for Windows©. Due to the limited number of rats in each group, non-parametric methods were used for statistical analysis. To compare the means of three or more groups, Kruskal–Wallis variance analysis was used to determine whether a statistical difference was present. The Mann–Whitney U Test was used to compare the means of two groups and to determine from which group the significant difference originated. A *p*-value of less than 0.05 was accepted as significant.

## 3. Results

**Figure 1:**
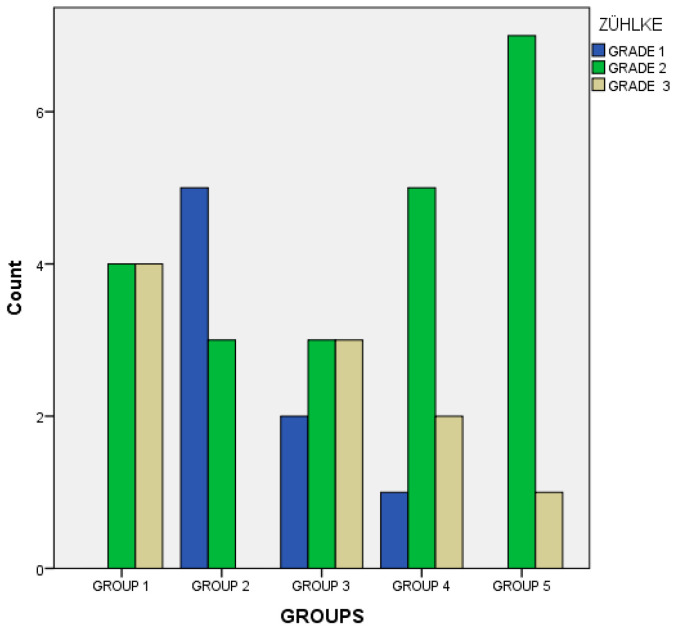
The histopathological grades of the groups.

IL1-β, TNF-α, E-selectin, and P-selectin levels were measured to evaluate the severity of inflammation and adhesion. Levels of the biochemical parameters and histopathological evaluation results are given in Table 2, while the histopathological changes between the groups are given in Figure 1.

The highest values of all of these four biochemical parameters were obtained in the control (C) group. There was a statistically significant difference (*p* < 0.05) between the control group and the other four groups.

When the levels of the biochemical parameters of the SF group were evaluated, there was a statistically significant difference between the sham, control, PRP, and TQ groups (*p* < 0.05). While the levels of all biochemical parameters were higher in the SF group than in the S, TQ, and PRP groups, they were significantly lower than those in the control group.

The values of the biochemical parameters of the PRP group were significantly higher than those of the S and TQ groups, but they were significantly lower than those of the C and SF groups (*p* < 0.05).

The levels of the biochemical parameters of the TQ group were the lowest among all the groups, which was statistically significant (*p* < 0.05).

After histopathological evaluation, the lowest Zühlke score was found to be from the S group, while the highest score was in the C group. There was also a statistically significant difference between the S group and C group, but there was no statistical difference between the S group and all other groups. The C group had the highest Zühlke score, and there was a statistically significant difference between the C, PRP, and TQ groups, but there was no significant difference between the C and SF groups.

## 4. Discussion

Abdominal surgery is the most common cause of intra-abdominal adhesions [25]. Inflammation of an organ (such as cholecystitis, appendicitis, and peritonitis), foreign objects left inside the abdomen after surgical operations, tuberculosis, inflammatory bowel diseases, bleeding into the peritoneal cavity, and pelvic inflammatory disease are among the other causes. Intra-abdominal adhesions may lead to mechanical ileus and require new operational procedures, such as a resection of the bowels, which may create stomas, impairing quality of life, or cause septic problems and eventually death due to perforation of the bowels [26]. To prevent these serious complications, it is important to understand the possible biochemical, histopathological, and immunological mechanisms of formation of intra-abdominal adhesions, as well as other types of mechanisms, and to conduct studies on preventive mechanisms.

Therefore, many experimental studies have been conducted to reduce or prevent intra-abdominal adhesions. Unfortunately, the majority of the items used in these studies cannot be used clinically. Furthermore, some treatments were accepted as effective in the beginning but were found to be ineffective in the long term.

Inflammation is a natural process of wound healing. It is the body’s first response when the peritoneum is injured due to surgery, trauma, ischemia, infection, or foreign bodies. Inflammation is characterized by the extravasation of blood serum and cellular elements [11]. Therefore, controlling the secretion of inflammatory mediators in the inflammatory–anti-inflammatory process should be the central focus if preventing adhesions is the main concern [12]. Many anti-inflammatory molecules have been studied for this purpose [4,5,27,28]. In this study, we used TQ and PRP, which have proven anti-inflammatory properties, as well as saline (SF), which has known adhesion-reducing properties.

We observed the lowest levels of biochemical parameters in the sham group. This result was expected because the S group was used to obtain the “normal” levels of the biochemical parameters evaluated in healthy animals. The levels of TNF-α, IL-1β, E-selectin, and P-selectin were the lowest in the TQ group but were higher than those in the S group. In a previous study by Ozden et al. [28], the use of TQ was found to be potentially effective in reducing adhesions. Since they only used microscopic and macroscopic adhesion valuations and did not use any biochemical parameters in their study, it was not possible to compare their results with those of our study in terms of biochemical parameters. The studies that investigated the potential effects of TQ on intra-abdominal adhesions are limited. In previous studies, authors most commonly evaluated histopathological scores and/or hydroxyproline levels [29,30]. Our study is the first to investigate the effects of TQ on the biochemical parameters of TNF-α, IL-1β, E-selectin, and P-selectin. There is no study to compare the parameters of these adhesions, but our results are compatible with previous inflammation studies on TQ, which have suggested that TQ can reduce or prevent the formation of intra-abdominal adhesions with its strong anti-inflammatory and antioxidant properties [28,29,30].

PRP has become an important substance used in many areas today due to its anti-inflammatory properties and growth factors. Due to its previously proven anti-inflammatory properties, it has been used in experimental animal models and is effective in both wound healing and the prevention of intra-abdominal adhesions [31,32]. Our study found that inflammation was significantly reduced in the PRP group compared to the control group. More specifically, all biochemical levels of the PRP group were lower than in the C and SF groups but higher than in the S and TQ groups. The fact that there is a significant difference between the PRP and control groups and the absence of a statistical difference with the sham group supports the idea that the use of PRP reduces intra-abdominal adhesions, which is in line with other studies in the literature.

Both PRP and TQ had good results compared to the C group in terms of biochemical parameters, but TQ had better results.

Irrigation with SF has been used for a long time, in both experimental studies and clinical practice, to reduce intra-abdominal adhesions. In experimental studies, SF was found to reduce intra-abdominal adhesions [33,34,35], and, in our study, all biochemical inflammation parameters were significantly lower in the SF group compared to in the control group, indicating agreement with the literature and demonstrating the adhesion-reducing properties of SF. However, in terms of biochemical parameters, SF was effective in reducing inflammation, but it was less successful than TQ and PRP.

Upon histopathological evaluation, as expected, there was a statistical relationship between the sham group and the control group. There were no significant differences between the other groups and the sham group, demonstrating that the treatments used are effective in recovery. Furthermore, the significant results between the control group and the TQ and PRP groups indicate that these two treatments are highly effective. However, although there was a noticeable difference between the SF and control groups, this difference is not reflected in the statistics, which reveals that the effectiveness in the SF group was weaker, as was similarly seen for the biochemical parameters. This result, especially the effectiveness of TQ, is in agreement with previous studies [28,30]. Studies investigating histopathological scores were not standardized; there were differences in the number of animals in the groups, the volumes and concentrations of the applied material, the purities of TQ, the use of black cumin oil or its derivatives (NSO, NSEE, or NSVO), the time intervals between the surgical procedure and the sacrifice of the animals, and the classifications of the histopathological scores [28,29,30,36,37] (Table 3).

As a result of this non-standardization, it is difficult to compare our histopathological scores with previous studies because of the different scoring systems used. However, we believe that the dose we used for TQ in our study, 1 mL (2 mg/mL), was effective. Nevertheless, more definite results for PRP, whose effectiveness has been demonstrated in our study once again, can be achieved with studies of larger samples and different dosage amounts, which may find increased effectiveness for different measures.

## 5. Conclusions

In this study, we compared the anti-adhesion formation effects of saline, thymoquinone, and platelet-rich plasma. Each treatment was found to be effective for intra-abdominal adhesions when assessed for biochemical inflammation parameters and histopathological adhesion scores. The most effective molecule in terms of reducing biochemical inflammation parameters was thymoquinone, but histopathological adhesion scores were promising in both TQ and PRP. We believe that further studies on the prevention of adhesion using thymoquinone, PRP, or a combination of the two should be conducted in the future for these molecules or materials containing these molecules so that they may become available and applicable in clinical practice.

## Figures and Tables

**Table 1:** Zühlke’s adhesion scale.

Grade	Description
1	Loose connective tissue, cell-rich, old and new fibrin, fine reticulin fibers
2	Connective tissue with cells and capillaries, few collagen fibers
3	Firmer connective tissue, fewer cells, more vessels, few elastic and smooth muscle fibers
4	Old, firm granulation tissue, cell-poor, hardly distinguishable serosal layers

**Table 2:** Scores of biochemical and histopathologic evaluations.

Groups	E-Selectin Mean (min–max) ng/mL	P-Selectin Mean (min–max) ng/mL	IL-1β Mean (min–max) ng/L	TNF-α Mean (min–max) ng/L	Zülhke Score Mean (min–max)
Group I: S (n:10)	65.50 ^a,b,c,d^ (62.04–68.96)	34.06 ^a,b,c,d^ (32.19–35.93)	61.69 ^a,b,c,d^ (57.59–65.58)	60.12 ^a,b,c,d^ (56.23–64.02)	1.38 ^a^ (1–2)
Group II: C (n:10)	157.18 ^a,e,f,g^ (120.21–184.24)	83.49 ^a,e,f,g^ (63.56–98.05)	164.86 ^a,e,f,g^ (123.26–195.32)	154.16 ^a,e,f,g^ (125.47–181.25)	2.5 ^a,f^ (2–3)
Group III: SF (n:10)	129.42 ^b,e,h,i^ (102.56–146.07)	68.52 ^b,e,h,i^ (54.06–77.50)	133.63 ^b,e,h,i^ (103.39–152.36)	125.09 ^b,e,h,i^ (102.36–141.78)	2.13 (1–3)
Group IV: PRP (n:10)	113.36 ^b,c,d,e^ (107.14–119.65)	59.86 ^b,c,d,e^ (56.51–63.25)	115.55 ^b,c,d,e^ (108.54–122.62)	113.98 ^b,c,d,e^ (106.98–121.06)	2.13 ^f^ (1–3)
Group V: TQ (n:10)	102.53 ^d,g,i,j^ (97.12–109.52)	54.02 ^d,g,i,j^ (51.14–57.80)	103.36 ^d,g,i,j^ (97.27–111.23)	95.25 ^d,g,i,j^ 80.25–105.03	2.13 ^g^ (1–3)

Values are presented as mean ± standard deviation. S: sham; C: control; SF: saline; PRP: platelet-rich plasma; TQ: thymoquinone. The differences between the following groups were all considered statistically significant (*p* < 0.05): ^(a)^ group I and group II; ^(b)^ group I and group III; ^(c)^ group I and group IV; ^(d)^ group I and group V; ^(e)^ group II and group III; ^(f)^ group II and group IV; ^(g)^ group II and group V; ^(h)^ group III and group IV; ^(i)^ group III and group V; and ^(j)^ group IV and group V.

**Table 3:** Volumes and concentrations of the material, sacrifice times, scoring systems used for histopathological evaluations, and results of different studies.

Study	Number of Animals in Each Group	Volume and Concentration of the Material	Sacrifice Time (days)	Histopathological Scoring System	Histopathological Result
Ozden et al. [28]	15	10 mg/kg TQ	21	Zühlke	TQ is histopathologically effective
Bozdag et al. [29]	10	10 mg/kg TQ *	15	Mazuji and Zühlke	TQ is histopathologically effective
Karatas et al. [30]	10	1 mL NSVO *** and NSEE ****	14	Evans	NSEE is histopathologically effective
Yilmaz et al. [36]	10	10 mg/kg TQ	7	Mazuji	TQ is histopathologically effective
Sahbaz et al. [37]	8	1 mL NSO **	8	Evans	NSO is histopathologically effective

* TQ: thymoquinone; ** NSO: *Nigella sativa* oil; *** NSVO: *Nigella sativa* volatile oil; **** NSEE: *Nigella sativa* ethanolic extract.

## Data Availability

All data included in this article are available from the corresponding author.
